# Ground-Based GNSS and Satellite Observations of Auroral Ionospheric Irregularities during Geomagnetic Disturbances in August 2018

**DOI:** 10.3390/s21227749

**Published:** 2021-11-21

**Authors:** Irina Zakharenkova, Iurii Cherniak, Andrzej Krankowski

**Affiliations:** 1 Space Radio-Diagnostic Research Center, University of Warmia and Mazury, 10-719 Olsztyn, Poland; iurii.cherniak@uwm.edu.pl (I.C.); kand@uwm.edu.pl (A.K.); 2 Pushkov Institute of Terrestrial Magnetism, Ionosphere and Radio Wave Propagation (IZMIRAN), 236006 Kaliningrad, Russia

**Keywords:** ionosphere, GNSS, ionospheric irregularities, ROTI, geomagnetic storm, auroral oval

## Abstract

The 25–26 August 2018 space weather event occurred during the solar minimum period and surprisingly became the third largest geomagnetic storm of the entire 24th solar cycle. We analyzed the ionospheric response at high latitudes of both hemispheres using multi-site ground-based GNSS observations and measurements onboard Swarm and DMSP satellites. With the storm development, the zones of intense ionospheric irregularities of auroral origin largely expanded in size and moved equatorward towards midlatitudes as far as ~55–60° magnetic latitude (MLAT) in the American, European, and Australian longitudinal sectors. The main ionospheric trough, associated with the equatorward side of the auroral oval, shifted as far equatorward as 45–50° MLAT at both hemispheres. The interhemispheric comparison revealed a high degree of similarity in a large expansion of the auroral irregularities oval towards midlatitudes, in addition to asymmetrical differences in terms of larger intensity of plasma density gradients and structures over the Southern auroral and polar cap regions. Evolution of the intense ionospheric irregularities and equatorward expansion of the auroral irregularities oval were well correlated with increases of geomagnetic activity and peaks of the auroral electrojet index.

## 1. Introduction

Formation and evolution of plasma density irregularities in the Earth’s ionosphere in response to space weather phenomena represent one of the fundamental problems of near-Earth plasma physics and a challenging task for operational models and space weather prediction. The most severe ionospheric irregularities occur primarily in the equatorial region, within a band of 20° S–20° N of magnetic latitudes (MLAT), and at the high-latitude region, above ~65° MLAT, which includes auroral and polar cap regions. These boundaries vary with time of day, season, sunspot number, and geomagnetic activity level. Geomagnetic storms are large disturbances in the Earth’s magnetosphere, usually measured through the ring current Dst index [1]. Intense geomagnetic storms are defined when the Dst index reaches –100 nT, whereas extreme storms (also called “superstorms”), are usually defined when Dst drops below –250 nT [2,3]. Geomagnetic storms are produced by enhanced solar wind–magnetosphere energy coupling through the magnetic reconnection mechanism between the interplanetary magnetic field (IMF) and the Earth’s magnetic field [4,5,6,7]. One of the most important phenomena of a geomagnetic storm is a magnetospheric substorm, in which a significant amount of energy derived from the solar wind–magnetosphere interaction is deposited into the auroral ionosphere and magnetosphere [8,9]. The energy input coming from the magnetosphere–ionosphere interaction in the form of enhanced electric fields, currents, and energetic particle precipitation perturbs the ionosphere through high-latitude ionization, Joule and particle heating, ion-drag forcing, and disturbed electric fields, producing ionospheric plasma irregularities and gradients enhancements [10,11,12]. The auroral oval encircling a magnetic pole represents a belt-like ionospheric region that receives the main part of the magnetospheric particle precipitation [13]. During geomagnetic disturbances, the auroral oval undergoes two types of change. The first is an enlargement of the auroral oval structure as a whole, and both the poleward and equatorward boundaries of the oval move equatorward. The second is repeated expansion and contraction of the width of the auroral oval, particularly in the midnight sector [13]. The location of the equatorward edge of the auroral oval depends on the energy of the precipitating particles and the magnetospheric electric and magnetic fields [14]. During magnetospheric substorms the boundaries of the auroral oval move. A magnetospheric substorm consists of three phases: growth, expansion, and recovery. At the growth phase of a substorm, dayside magnetic field reconnection between the southwardly directed IMF and the geomagnetic field increases the number of open field lines—as a result, the polar cap expands due to the added open flux, and the auroral oval migrates equatorward to lower latitudes. In the expansion phase of a substorm, energy stored in the magnetotail is explosively released into the ionosphere [15]. The aurora suddenly brightens and expands poleward as the magnetotail performs a dipolarization. The energetic particle precipitation enhances the conductivity in the ionosphere, which causes a sudden enhancement of the auroral electrojets. During the third, recovery, phase of a substorm, the intensity of the auroral emissions reduces. The auroral oval repeats expansion and subsequent contractions of its width, particularly in the nighttime sector. The expansion and contraction of the auroral oval width occur during the expansion and recovery phases of the substorm, respectively. Auroral particle precipitation creates highly structured enhancements and gradients of the ionospheric plasma density. Such ionospheric irregularities occurring during intense geomagnetic storms can cause rapid phase fluctuations in Global Positioning Satellite System (GNSS) signals. For several decades, ground-based GNSS measurements have been used for investigation and regular monitoring of the occurrence of ionospheric irregularities. At high latitudes, phase scintillations of GNSS signals increased drastically along an L shell when the ground-based station was under the auroral oval [16]. Using GPS observations from 11 high-latitude stations, Aarons [17] also noted that phase fluctuation activity has a daily pattern mainly controlled by the motion of the receiver location into the auroral oval. In general, the zone of the intense ionospheric irregularities as defined by GNSS phase fluctuations changes in size and correlates with changes in the auroral oval width [17,18]. The high-latitude ionospheric irregularities that are detected at trans-ionospheric paths are thought to be in both the E and the F layers with a combination of structured hard and soft electron precipitation and coupling initiating the turbulent activity [19]. It is important to note that auroral ionospheric irregularities can be observed far away from the auroral oval zone. Their lifetime is inversely related to the ionospheric conductivity. Therefore, they can be transported considerable distances from their source by the action of high-latitude electric fields. In a two-cell convection pattern which prevails for IMF southward conditions, the plasma, and hence the irregularities, are convected equatorward from the auroral oval towards the cusp region, and then across the polar cap to the nightside auroral oval [20]. The polar cap patches are 100–1000 km scale segmented regions of enhanced ionospheric F-layer plasma density, convecting across the polar cap in a generally anti-sunward direction [21]. The dynamics of such irregularities is controlled by the IMF [22]. The patches often contain a smaller scale structure, particularly on the edges, as determined from scintillation effects on GNSS signals passing through regions of ionospheric irregularities of scale size around tens to hundreds of meters [23]. Thus, both the ionospheric irregularities embedded in polar patches and the auroral irregularities produced by intense auroras, result in scintillations—rapid fluctuations of radio wave amplitude and phase that may adversely affect performance and operational capabilities of radio communication and navigation satellite systems [24]. With a storm development, the zone of the intense high--latitude ionospheric irregularities may largely expand equatorward from auroral/subauroral latitudes and, occasionally, affect midlatitudes (down to 50–55° MLAT in both hemispheres). For example, observations of auroral ionospheric irregularities at midlatitudes (40–50° N) were reported in Europe during the Halloween 2003 superstorm [25] and in North America for the St. Patrick’s Day 2015 storm [26,27] and caused serious degradation of the kinematic GPS positioning performance for the ground-based segment [28]. In this paper, we examine an occurrence and evolution of the storm-induced auroral ionospheric irregularities during a space weather event of 25–26 August 2018. This interesting event was classified as the third largest geomagnetic storm of the entire 24th solar cycle (in terms of the Dst minimum excursion) after the March 2015 and June 2015 geomagnetic storms. Initially, it was expected to only be a minor geomagnetic storm, but surprisingly transformed into a strong geomagnetic storm at the end of the 24th solar cycle [29,30].

## 2. Materials and Methods

This study was based on processing and analysis of measurements provided by multiple networks of the ground-based GNSS receivers (6000+). We analyzed an occurrence and evolution of the ionospheric irregularities using a specific GPS-based index—ROTI (Rate of TEC Index change)—that was originally proposed by Pi et al. [31]. This technique is now widely utilized for detection and specification of the ionospheric irregularities at regional and global scales [32,33,34,35,36]. This index characterizes intensity and sharpness of the GPS/GNSS phase fluctuations caused by ionospheric irregularities and by strong spatial gradients of TEC. The ROTI values were calculated for every visible GPS and GLONASS satellite (elevation above 20°) over a ground-based GNSS station. The ROTI values were referred to the intersection (ionospheric pierce point—IPP) of the line-of-sight with the thin ionospheric layer at 350 km altitude. Further, the derived ROTI values with IPP coordinates were averaged into a 0.5° latitude/longitude grid to create the global ROTI maps with a high spatio-temporal resolution, in addition to the daily ROTI maps for the Northern Hemisphere (for more details on the mapping technique, see [37,38]).

To examine the intensity of the storm-induced ionospheric irregularities at different altitudes of the topside ionosphere, we used observations from several Low-Earth-Orbiting satellites. First, we analyzed observations provided by the European Space Agency (ESA) Swarm mission of three identical satellites—Swarm A, Swarm B, and Swarm C—operating at polar orbits of ~88° inclination. The Swarm B satellite had a higher orbit of ~510 km altitude, whereas the two other satellites were flying in a tandem (separation of ~1° in space and ~9 s in time) at an orbit altitude of ~450 km. We used in situ electron density (Ne) data from the Langmuir probe instrument. Second, we used in situ ion density (Ni) measured by the Ion Velocity Meter instrument onboard DMSP (Defense Meteorological Satellite Program) satellites at a much higher orbit altitude (~860 km). As of 26 August 2018, the local times of the ascending and descending nodes were ~15.8 LT/~3.8 LT for the F16 satellite, and ~18.6/~6.6 LT for the F17 satellite, respectively.

## 3. Results and Discussion

### 3.1. The 25–26 August 2018 Geomagnetic Storm

**Figure 1:**
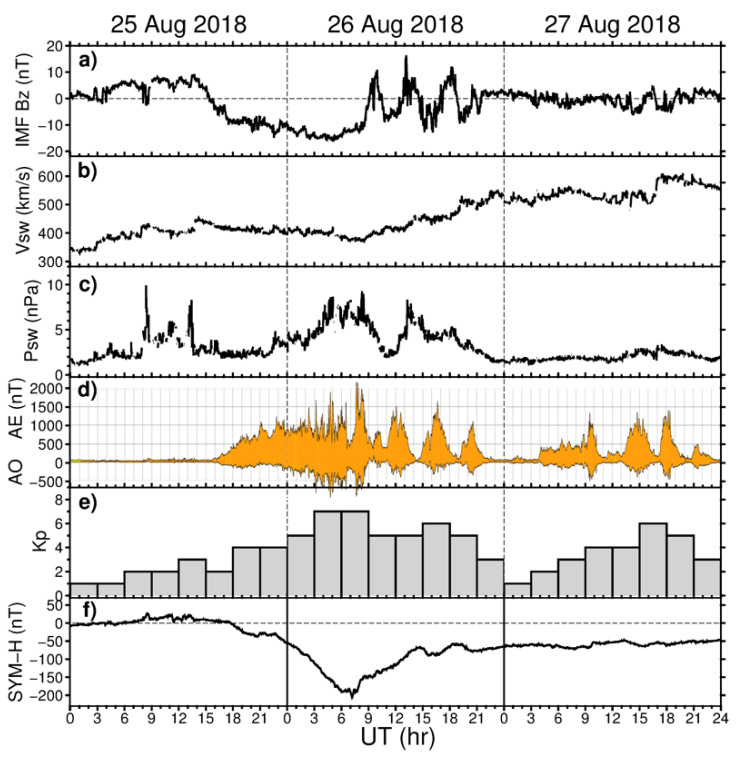
Geomagnetic conditions during 25–27 August 2018: (**a**) IMF Bz component, (**b**) velocity and (**c**) dynamic pressure of the solar wind, (**d**) auroral electrojet index AE, (**e**) Kp index, and (**f**) SYM-H index.

The 25–26 August 2018 geomagnetic storm is considered as the third largest geomagnetic storm of the 24th solar cycle in terms of the Dst minimum excursion (Dst minimum reached −174 nT). This storm was produced by a slow CME on 20 August 2018, which arrived at the Earth’s magnetosphere on 25 August 2018. Figure 1 shows variations of the major geophysical parameters during 25–27 August 2018. The The IMF southward turningoccurred after 14 UT on 25 August 2018 (Figure 1a). The IMF Bz remained steadily negative for more than 17 h from 15:30 UT on 25 August until 09:00 UT on 26 August, and the peak Bz component reached −16 nT near 05 UT on 26 August 2018. During this period, the main phase of the storm developed from ~18 UT on 25 August and the SYM-H index dropped to a minimum of −206 nT at ~07:10 UT on 26 August 2018 (Figure 1f). The AE (auroral electrojet) index increased above 500 nT after 18 UT on 25 August 2018, and the AE peaks exceeded 1500–2000 nT during 02–09 UT on 26 August 2018. During the main phase of storm, the Kp index reached 7+ (Figure 1e), and the storm was classified as a strong, G3-level storm in the NOAA Space Weather scale.

### 3.2. Ground-Based GNSS ROTI Observations

**Figure 2:**
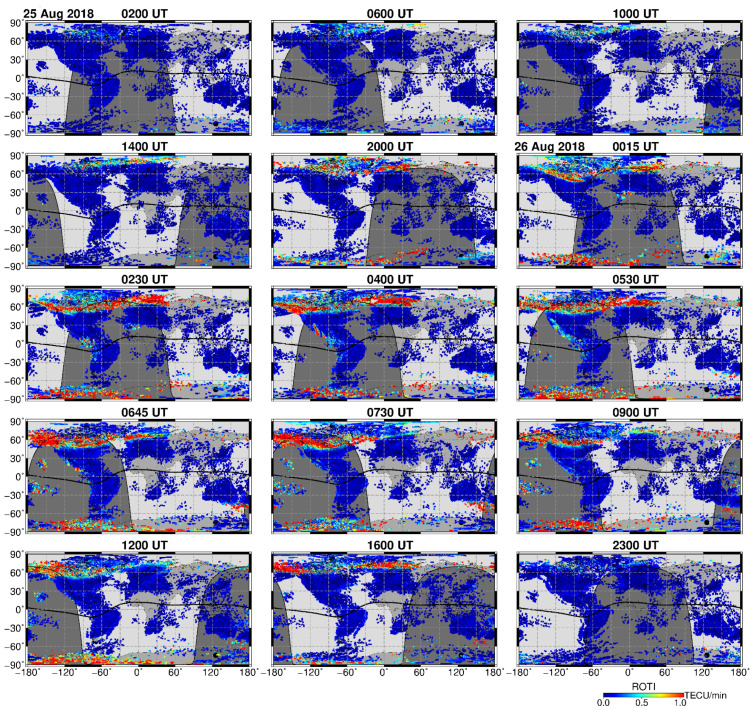
Global GNSS ROTI maps for selected times on 25–26 August 2018. The thick black line marks the magnetic equator, the grey shaded area shows nighttime. High ROTI values (intense red color) depict severe ionospheric irregularities occurrence at equatorial and auroral zones.

We investigated an occurrence and development of the storm-induced ionospheric irregularities at a global scale that were identified through their impact on the received GNSS signals using the multi-site ground-based GNSS ROTI observations. Figure 2 presents an overview of the occurrence of ionospheric irregularities at a global scale before and during the storm’s main phase development. The first ROTI maps for 25 August 2018 corresponding to the pre-storm period show rather a typical situation, with an absence or very low intensity of ionospheric irregularities (small ROTI magnitudes marked by dark-blue color) practically everywhere around the globe with some intensification of the ionospheric irregularities at high latitudes close to the magnetic poles. At 20 UT on 25 August, with an increase in auroral activity (AE index exceeded 500 nT after ~19 UT), the global ROTI map depicts a significant intensification of the ionospheric irregularities’ occurrence at high latitudes of both hemispheres. Later, as the storm developed, the belt-like area with the strong ionospheric irregularities of auroral origin largely expanded in size and moved equatorward towards midlatitudes as far as ~60° MLAT in the American, European, and Australian longitudinal sectors. Additionally, one can note an occurrence of the intense equatorial ionospheric irregularities at low latitudes of the African, American, and Pacific regions.

**Figure 3:**
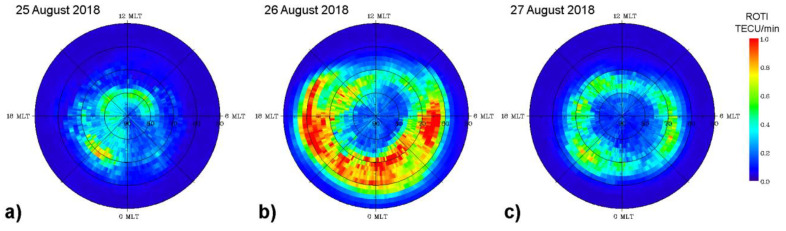
Daily MLT-MLAT ROTI maps for the Northern Hemisphere for (**a**) 25 August, (**b**) 26 August, and (**c**) 27 August 2018. The maps cover 50–90° N MLAT with 10° latitude circles; magnetic local noon/midnight is at the top/bottom, and dusk/dawn is toward the left/right. Blue color corresponds to the absence or very weak ionospheric irregularities, red color—severe ionospheric irregularities occurrence.

Figure 3 presents a sequence of the IGS daily ROTI maps constructed for the three consecutive days of 25–27 August 2018. These daily ROTI maps were created according to the approach described in detail in [37]. The daily ROTI maps represent an overall pattern of the spatial distribution of the ionospheric irregularities over the Northern Hemisphere high and middle latitudes. For this type of map, we processed ROTI data from a representative set of ~700 permanent GPS stations instead of the whole dataset of the 6000+ stations, and the final result was visualized in the form of the ROTI map in the polar view projection. The ROTI map for each day represents the ionospheric irregularities’ distribution specified by ROTI as a function of a magnetic local time (MLT) and corrected magnetic latitude (MLAT) within a 00–24 MLT time frame and 50–90° N MLAT range. The value in every cell is calculated by averaging of all ROTI values covered by this cell area and is proportional to the irregularities’ occurrence probability in the current sector.

For the relatively quiet day of 25 August 2018 (Figure 3a), the area affected by ionospheric irregularities was within 80° MLAT on dayside and 70–72° MLAT at nightside when the sector with maximum intensity of ROTI corresponded to 19–22 MLT. For the day of the main phase of the geomagnetic storm, 26 August 2018 (Figure 3b), the daily ROTI map shows a dramatic difference in comparison to the previous day. In particular, the auroral ionospheric irregularities had high intensity (ROTI values exceeded 1.0 TECU/min), and their spatial distribution formed a clear oval-like shape (similar to an auroral oval’s form seen in the satellite-based UV observations) largely expanded towards middle latitudes. The auroral irregularities’ oval expanded equatorward as far down as ~55° MLAT for the night-time sector and 60–65° MLAT for the dayside. Figure 3c shows the corresponding daily pattern of the high-latitude ionospheric irregularities for the storm’s recovery phase on 27 August 2018. This day still had several intervals with increased auroral activity where the AE peaks exceeded 1000 nT. One can see a noticeable decrease in the ionospheric irregularities’ intensity specified by ROTI with the maximal averaged values around 0.4–0.6 TECU/min. The irregularities’ oval shape is still recognizable for this less disturbed day, but it shrank essentially in size and its equatorward edges were detected near 65° MLAT in the night-time sector and near 75° MLAT on the dayside. The sector with maximum intensity of ROTI corresponded to 19–22 MLT, similar to the pre-storm day.

**Figure 4:**
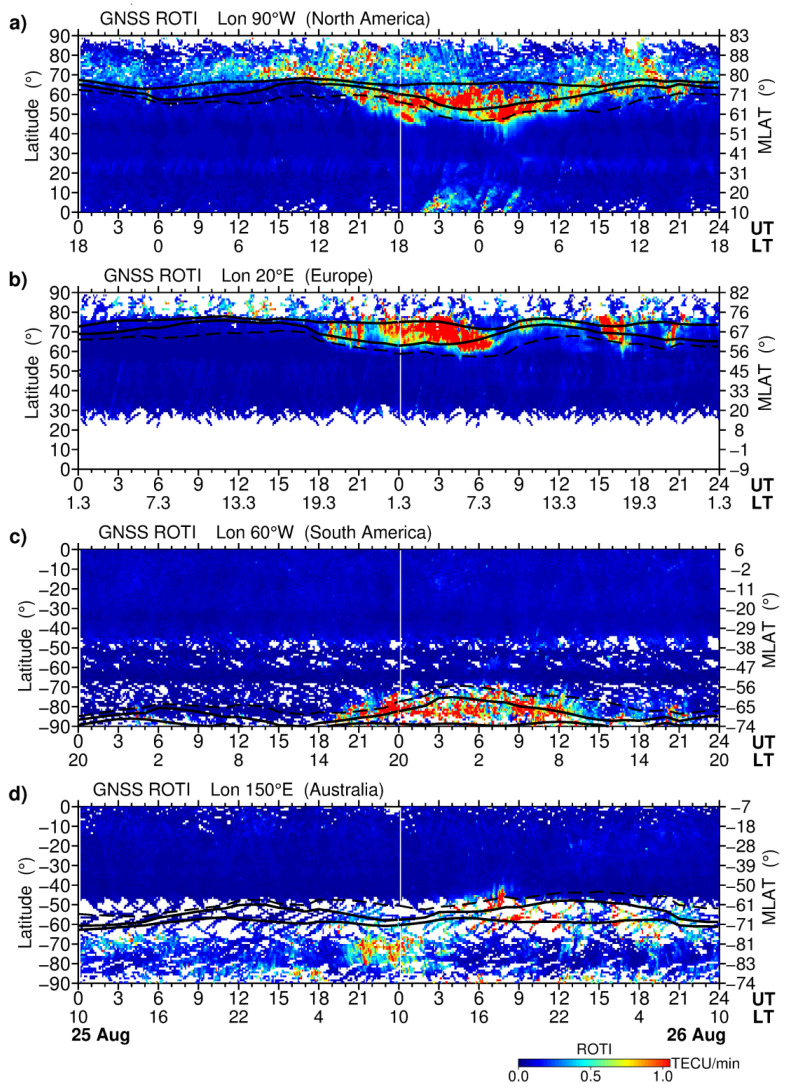
North–south cross-sections (keograms) representing the spatio-temporal features of the storm-induced ionospheric irregularities as detected in GNSS ROTI along (**a**) 90° W, (**b**) 20° E, (**c**) 60° W, and (**d**) 150° E longitudes in the Northern and Southern Hemispheres during 25–26 August 2018. Vertical axes show (left) geographic and (right) geomagnetic latitudes, horizontal—UT and LT time. White color depicts empty cells due to lack of actual observations. The auroral oval boundaries predicted by the Feldstein–Starkov model are marked by the black solid lines for the poleward and equatorward boundaries, and by a dashed line for the diffuse boundary.

Figure 4 compares a temporal evolution of the storm-induced ionospheric irregularities summarized in the form of the north–south cross-sections (keograms) of the GNSS ROTI maps along particular longitudes in the American, European, and Australian sectors during 25–26 August 2018. These keograms, plotted as a function of geographic latitude and UT/LT time, present the averaged ROTI values across a narrow longitudinal range (±5°) around the considered longitude. To show the auroral oval prediction for this geomagnetic storm, we include simulation results of the Feldstein–Starkov empirical model of the auroral oval. The Feldstein–Starkov empirical model calculates the size and location of the auroral oval, which includes the poleward, equatorward, and diffuse auroral boundaries, as a function of the planetary Kp index [39,40]. These auroral oval boundaries extracted for the considered longitudes were superimposed on the GNSS ROTI keograms.

For North American longitudes (Figure 4a), the intense ionospheric irregularities started to develop at high latitudes (80–85° MLAT) after ~13 UT on 25 August 2018. In the next hours, with the progression of the main phase of the storm and further rise of the AE index to ~1000 nT, the zone with intense auroral irregularities expanded equatorward and reached ~50° N (60° MLAT) at 00–02 UT on 26 August 2018 and during the period of minimal Dst excursion at 06–08 UT. One can note that auroral irregularities form a belt-like zone expanded as a whole toward midlatitudes, whereas at higher latitudes close to the magnetic pole, a considerably smaller intensity of the GNSS ROTI values corresponding to the polar cap region was registered. Comparison with the model-derived auroral oval boundaries revealed that the observed auroral ionospheric irregularities expanded far more equatorward than those of the model predictions. Moreover, for this longitudinal sector, the storm-induced irregularities of equatorial origin occurred during 02–09 UT and extended as far northward as ~10–20° MLAT. For the European sector (Figure 4b), the most intense auroral irregularities were registered during the main phase of the storm from ~18 UT on 25 August until ~08 UT on 26 August 2018. The auroral irregularities zone expanded as far equatorward as ~60° N (55–60° MLAT). In the storm’s recovery phase, the auroral irregularities demonstrated a rapid decrease in intensity and a poleward motion of this zone during local daytime hours. Two intervals with new peaks in high ROTI values were observed near 16 UT and 19 UT that corresponded to the peaks with the short-term AE index increase above 1000 nT at the same times. For the Southern Hemisphere, quite a limited amount of area is covered by the ground-based networks of GNSS receivers. Here, we selected longitudes across the South America continent and Australia to obtain the best coverage by the ground-based GNSS observations. Figure 4c shows the occurrence and evolution of the ionospheric irregularities along 60° W longitude in South America. The GNSS ROTI observations detected the auroral irregularities developed mainly during the main phase of the storm from ~19 UT on 25 August until ~13 UT on 26 August 2018 that corresponded to the local dusk, nighttime, and dawn conditions. This zone was expanded as far equatorward as ~70° S (55° MLAT), thus exceeding the model-predicted equatorward and diffusive aurora boundaries. Figure 4d shows ROTI values extracted along 150° E longitude in the Australian region. Here, we have sparse GNSS data coverage, especially between the Australia and Antarctica continents. Because the south geomagnetic pole shifted equatorward (~74° S), for this longitudinal sector (150° E) the major part of the extended auroral oval is expected to be observed over this region of sparse data coverage. We can note that during the main phase of storm (18–08 UT) that corresponded to the local daytime conditions, the most intense ionospheric irregularities were observed in the polar cap region, poleward from the model-predicted poleward boundary of the auroral oval. During the recovery phase, after ~08 UT on 26 August 2018 with the progression into local dusk and nighttime sectors, the ionospheric irregularities were detected at much lower latitudes, close to the Australia region, as far equatorward as 45–50° S (55–60° MLAT).

### 3.3. Satellite Observations

The ground-based GNSS observations serve as an excellent tool for continuous monitoring of the ionospheric electron density and ionospheric irregularities over areas with dense networks of the ground-based receivers. However, these ground-based GNSS measurements do not allow the determination of an altitudinal extent or localization of the detected ionospheric irregularities along a line-of-sight from a ground-based receiver to a GNSS satellite. It is generally believed that ionospheric irregularities, detected with ground-based GNSS measurements, correspond to the ionospheric F layer irregularities phenomena, which occurred in a region with maximal electron density in a vicinity of the F2 peak altitude (~250–400 km). Clearly, ionospheric irregularities can propagate to much higher altitudes, especially in the polar ionosphere along the magnetic field lines. In these circumstances, only satellites can provide reliable observations of plasma density structures at specific altitudes of the topside ionosphere on a global scale, but only as a one-dimensional cut of the ionosphere along the particular satellite orbit.

**Figure 5:**
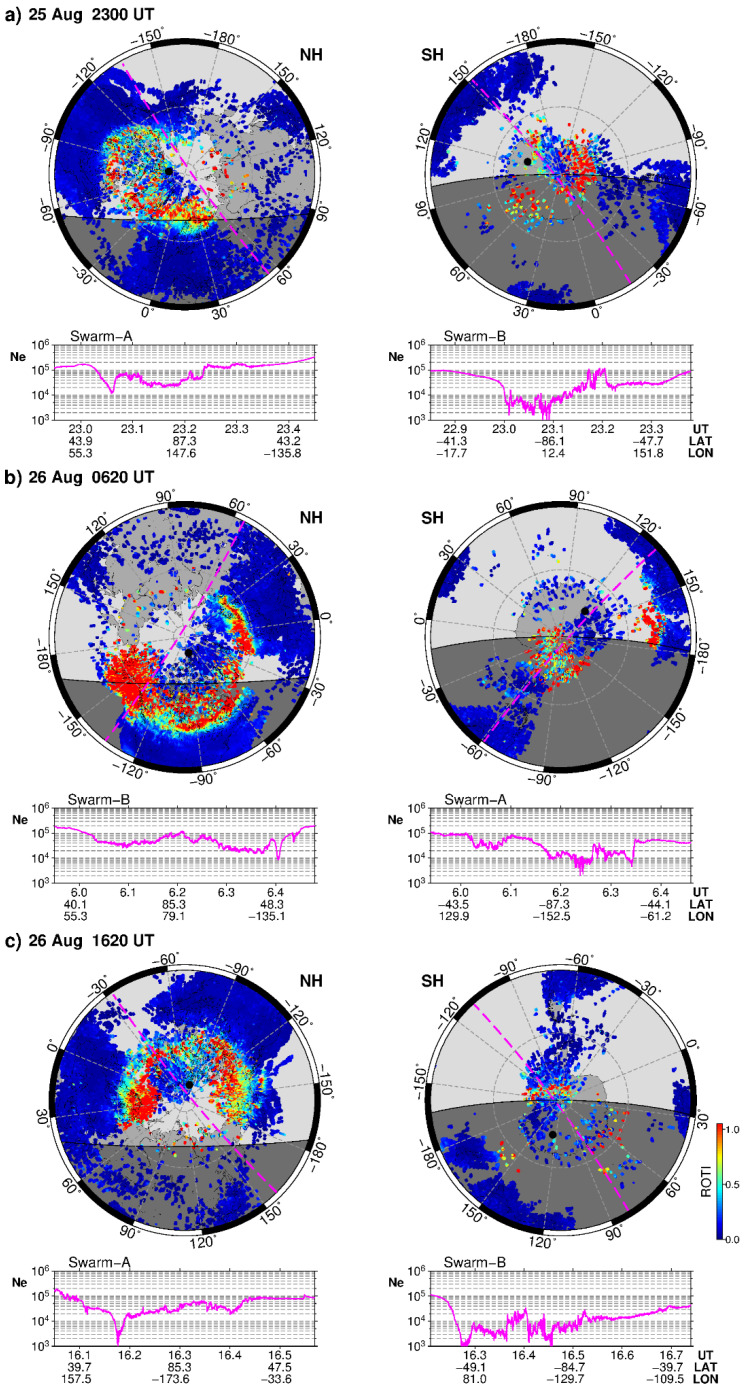
Two-dimensional GNSS ROTI maps in a polar view projection illustrating development of the auroral irregularities oval over the Northern (NH) and Southern (SH) Hemispheres for (**a**) 23:00 UT on 25 August, (**b**) 06:20 UT on 26 August, and (**c**) 16:20 UT on 26 August 2018. The maps cover 30°–90° N/S with 30° latitude/longitude grid. The grey shaded area shows nighttime, the maps are rotated with local midnight to be at the bottom. Black dot depicts location of the geomagnetic poles, the dashed magenta line shows the projection of the Swarm satellite overpass. Bottom panel of each plot shows variability of in situ electron density (Ne) along the Swarm overpass together with information about corresponding UT and geographic coordinates.

In Figure 5, we present a comparison of the high resolution two-dimensional GNSS ROTI maps plotted in geographical coordinates with a polar projection over the Northern and Southern Hemispheres for several representative time intervals of the geomagnetic storm. For each map, we found the corresponding orbit of the Swarm A or Swarm B satellites that overpassed the polar region of the considered hemisphere and included plots with variability of the in situ electron density along this overpass (~500 km orbit altitude). Figure 5a shows results for 23 UT on 25 August 2018, at the beginning of the main phase of the geomagnetic storm. At that time, the AE index rose to ~1000 nT and plasma irregularities detected by ground-based GNSS observations already formed an oval-like structure over the Northern Hemisphere pole, with a wider part extended towards the dusk and nighttime sectors. In the Southern Hemisphere, the intense ionospheric irregularities were observed over a narrower zone, mainly over the over the Antarctica continent. The Swarm in situ plasma density measurements show an asymmetry in the background density level for the Northern and Southern Hemispheres, when density in the summer sunlit hemisphere is noticeably higher than that for the winter (southern) one. The smooth parts of the Swarm Ne plots correspond to the part of the satellite tracks over midlatitudes, and the central part with rapid density variations represents plasma density structuring across the polar region. Over the Southern Hemisphere polar area, the plasma density variability was larger, and dropped to lower values than those at the Northern pole area. Figure 5b presents two GNSS ROTI maps for 06:20 UT on 26 August 2018, close to the peak of the storm’s main phase when the Kp index reached its maximal value of 7+ during this event and the AE index was 1000–1500 nT. At that time, in the Northern Hemisphere, the irregularities oval was well formed and expanded towards midlatitudes of the North America and Europe regions; the widest area (~25° in latitude span) covered by intense auroral irregularities was detected over Alaska in the local dusk sector. In the Southern Hemisphere, the auroral irregularities zone also expanded towards lower latitudes reaching southern Australia—this region was geomagnetically conjugated with the concurrent intensification of the auroral irregularities over Alaska in the Northern Hemisphere. The Swarm in situ plasma density observations during that time revealed rapid density variations across the polar regions, and noticeable expansion in the size of the entire zone of auroral irregularities and the main ionospheric trough, associated with the equatorward side of the auroral oval, moved to lower latitudes. The main ionospheric trough is also considered as an ionospheric footprint of the plasmapause; it divides the plasmasphere-maintaining ionosphere (closed magnetic flux tubes) from the precipitation-maintaining auroral ionosphere. Figure 5c presents results for 16:20 UT on 26 August 2018, a period in the storm’s recovery phase with one of the substorm activity intensification peaks (AE burst-like rise from ~100 nT to 1000–1500 nT at 15–18 UT). We observed a well-developed oval of the ionospheric irregularities expanded from the auroral zone down to ~60° N in North America and Europe. The oval’s widest parts (~15–20° in latitude span) were found at local dusk and dawn sectors. In the Southern Hemisphere, the irregularities’ oval is seen only partially because its most part was above the ocean area, due to a lack of coverage by the ground-based GNSS stations. The in situ observations onboard the Swarm satellites demonstrated that the level of the plasma density structuring with steep gradients was much larger over the Southern polar region (winter season, non-sunlit at night) than that of the Northern one. At that time in both hemispheres, the main ionospheric trough had a very deep minimum, which was quite a large distance between well-defined equatorward and poleward walls, and its equatorward wall moved towards midlatitudes as far down as ~52° N (~45°MLAT) and ~34° S (~45°MLAT).

**Figure 6:**
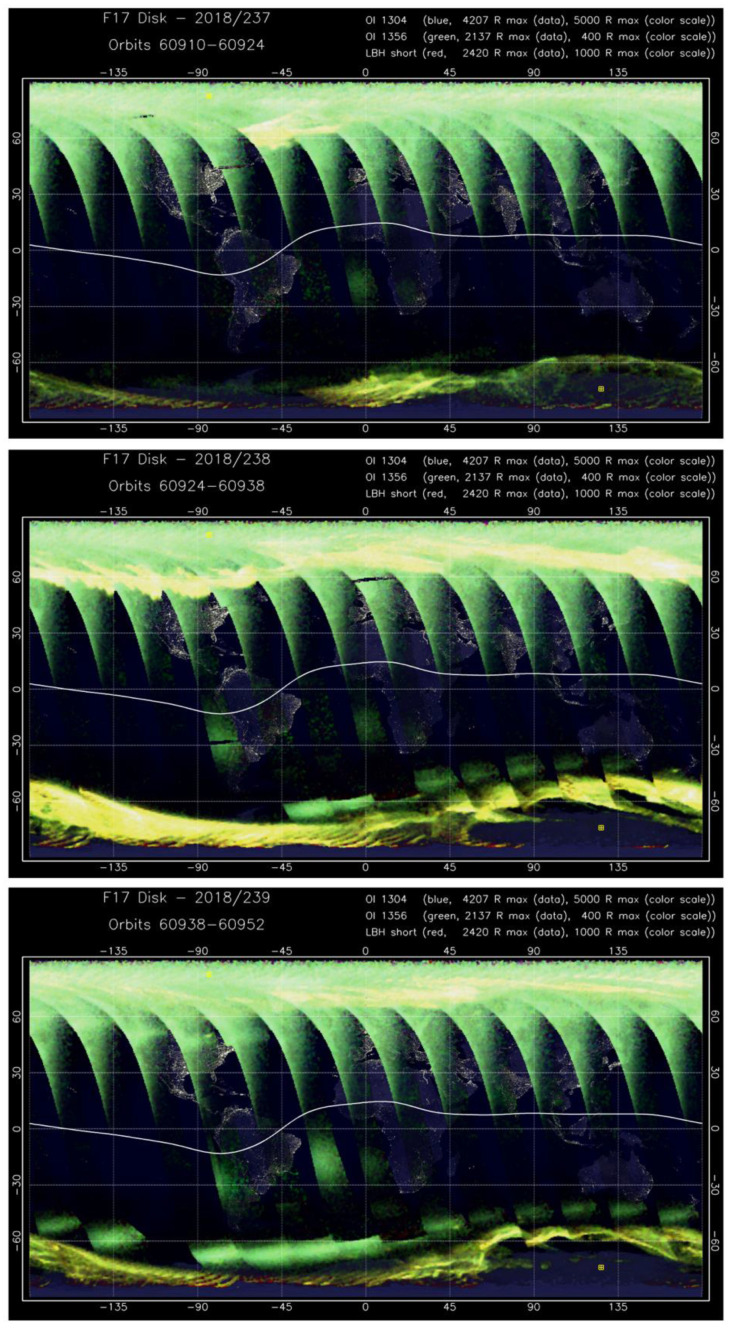
L1B Daily Summary Images from the Special Sensor Ultraviolet Scanning Imager (SSUSI) sensor onboard DMSP F17 satellite with a map projection of nightside disk data with nighttime solar zenith angles for 25–27 August 2018 (Day of Year 2018/237–2018/239) (https://ssusi.jhuapl.edu/images_daily_l1b (accessed on 16 November 2021)). For 25 August (top plot), the first DMSP F17 pass was near 80° W, next passes progressed to the left.

For the case of the August 2018 geomagnetic storm, we also examined the optical observation of the ionosphere by the Special Sensor Ultraviolet Spectrographic Imager (SSUSI), which measures far ultraviolet emissions in five different wavelength bands (HI 121.6 nm, OI 130.4 nm, OI 135.6 nm, N2 LBHS (140–150 nm) and N2 LBHL (165–180 nm)) from the Earth’s upper atmosphere [41]. These channels capture the main auroral UV emissions. Figure 6 presents daily summary SSUSI images for all nightside passes of the DMSP F17 satellite during 25–27 August 2018. It clearly shows a difference between the Northern and Southern Hemispheres in intensity of emissions at high latitudes. For 25 August (Figure 6, top), the first DMSP F17 pass was near 80° W, and the following passes progressed to the left. During the first passes, rather narrow belts of auroral emissions were observed at high latitudes; the most pronounced intensification of the auroral emissions was registered in the conjugate regions of both hemispheres when the satellite overpassed the African and Atlantic sectors (50° W–45° E) at the end of the day, when the main phase of the geomagnetic storm began. For 26 August (Figure 6, middle), the first pass started again near 80° W, and as the storm developed, the belts of bright auroral emissions significantly enlarged in size (~10–15° in latitudinal span) and moved equatorward in both hemispheres reaching ~50° MLAT. The auroral emissions occurred at high-to-middle latitudes of North America and Europe, and close to South America and Australia in the Southern Hemisphere. The spatial extent of the intense auroral emissions was larger in the Southern Hemisphere. These UV observations agreed well with results of the ground-based GNSS observations of the intense ionospheric irregularities and their evolution in time and space. During the recovery day of 27 August (Figure 6, bottom), SSUSI images still captured the bright auroral emissions at high latitudes of both hemispheres, but they were noticeably narrow in a latitudinal span compared with those of the main phase day. The equatorward edge of the auroral emissions moved poleward, but a bright aurora was still registered at both hemispheres across all longitudinal sectors.

**Figure 7:**
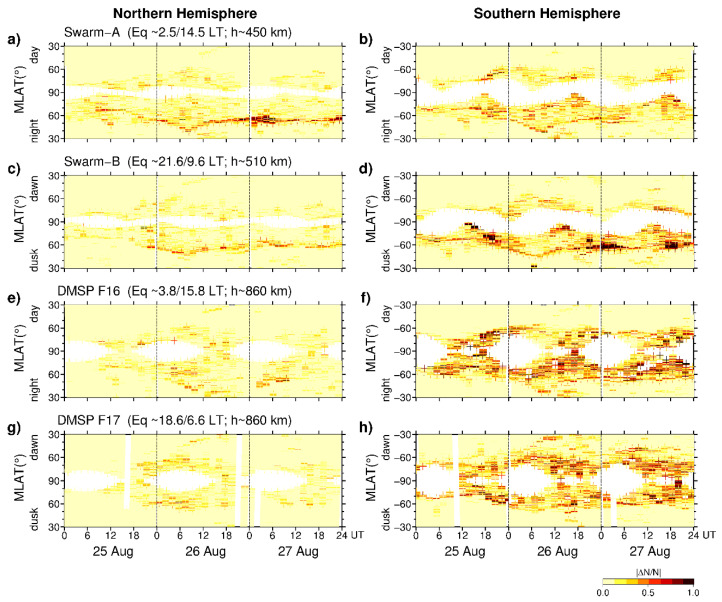
Satellite-based in situ density variations |∆N/N| plotted as a function of geomagnetic latitude (the y axis) and UT time (the x axis) during 25–27 August 2018 for the (**left**) Northern and (**right**) Southern Hemispheres for (**a,b**) Swarm-A, (**c,d**) Swarm-B, (**e,f**) DMSP F16, and (**g,h**) DMSP F17 satellites. Continuity leaps (white areas close to 90° N/S MLAT) appear due to satellite pass displacement from the magnetic poles.

During this space weather event, several satellites of the Swarm and DMSP missions overpassed high latitudes region of both hemispheres and encountered storm-induced auroral irregularities at altitudes of ~500 and ~860 km, respectively. Both missions provided actual measurements of in situ plasma density variation along an orbit. Further, in our analysis, all satellite passes were divided into duskside/dawnside or night/day parts by separation of their ascending and descending nodes. Then, the time series of the Swarm and DMSP in situ plasma density values with 1 Hz rate were processed using a running window technique to derive relative density variation |∆N/N| normalized to N, where N is mean density value calculated on a 15 s window interval (~1° in space). Figure 7 presents an overview of the relative plasma density variations for all satellite passes over the Northern and Southern Hemispheres during 25–27 August 2018. Results are presented as a function of geomagnetic latitude (MLAT) and UT time. Continuity leaps, seen as white areas close to 90° N/S, appear due to satellite pass displacement from the geomagnetic poles; because s satellites did not pass exactly overhead the geomagnetic pole, their observations could not cross all geomagnetic latitudes till 90° N/S. Moreover, some continuity leaps can occur due to the absence of actual measurements along a track. Light yellow color marks the location of very weak plasma density gradients. We can note that with the storm development starting on 25 August 2018, the pronounced intensification of plasma density gradients was registered at high latitudes of the Northern Hemisphere in the Swarm A and Swarm B observations (Figure 7a,c), and the largest ones were associated with an equatorward move and deepening of the main ionospheric trough at the night and dusk sides of the satellite passes during the three considered days. The main feature is the large interhemispheric difference in intensity and magnitude of the ionospheric plasma density gradients between Northern and Southern Hemispheres (left and right panels of Figure 7). In the Southern Hemisphere, the intensity of plasma density gradients was significantly higher than that registered by two Swarm satellites at lower altitudes of ~500 km; this interhemispheric difference was even more pronounced at DMSP orbit altitudes of ~860 km.

## 4. Conclusions

We analyzed the response of the high-latitude ionosphere to the 25–26 August 2018 geomagnetic storm that occurred at the end of the 24th solar cycle. According to the space weather predictions, it was expected to only be a minor geomagnetic storm, but surprisingly transformed into a strong geomagnetic one, and was the third largest storm of the entire solar cycle. The prominent feature of this response was the development of the storm-induced ionospheric plasma density irregularities of auroral origin that were detected using a combination of the ground- and space-based observations. The high spatio-temporal resolution GNSS ROTI mapping approach provides a very detailed specification of the storm-induced ionospheric plasma density irregularities at small to medium scales. Using this approach, we successfully demonstrated how during the major space weather event the ionospheric irregularities’ oval expanded largely in size towards midlatitudes with a simultaneous increase in the irregularities’ intensity. This was associated with an increase in auroral activity caused by auroral particle precipitation and the generation of plasma instabilities related to the intensification of electric fields during geomagnetic disturbances.

The major results can be summarized as follows:

1. The auroral ionospheric irregularities’ oval formed after the geomagnetic storm onset and progressively expanded in size and location towards midlatitudes with the storm development. In its widest part between poleward and equatorward boundaries, the oval was 15–25° in latitudinal span.
2. The zones of intense ionospheric irregularities of auroral origin moved as far equatorward as ~55–60° MLAT in the American, European, and Australian longitudinal sectors.
3. The main ionospheric trough, associated with the equatorward side of the auroral oval, shifted as far equatorward as 45–50° MLAT in both hemispheres.
4. Joint analysis of the ground-based GNSS ROTI together with the Swarm in situ observations showed that topside plasma density irregularities and the main ionospheric trough locations along satellite tracks were consistent with the irregularities oval location specified by GNSS ROTI.
5. The intensity of the ionospheric plasma density irregularities detected by the GNSS ROTI techniques at high and middle latitudes had comparable magnitudes with those observed during the largest geomagnetic storm of this solar cycle, the St. Patrick’s Day storm in March 2015 [26,28].
6. In the topside ionosphere, the strong plasma density gradients were registered at altitudes of 500 and 860 km onboard Swarm and DMSP satellites during the main and recovery phases of the storm. The satellite observations revealed a large interhemispheric asymmetry in the gradients’ intensity prevailing in the Southern (winter) Hemisphere.
7. We report a good consistency between the occurrence of ionospheric irregularities and locations derived from the ground-based GNSS observations and LEO satellite measurements with the auroral activity captured in FUV observations by the DMSP F17 SSUSI instrument.

The presented results emphasize the importance of continuous space weather monitoring by multiple ground-based and space-borne sensors because, even during the deep solar minimum conditions, major geomagnetic storms can occur and produce an unexpectedly strong response of the Earth’s ionosphere. This poses a significant space weather threat to real-time precise positioning and navigation systems relying on GNSS and radio wave propagation.

## Data Availability

The Swarm data were obtained from the European Space Agency (http://earth.esa.int/swarm (accessed on 16/11/2021)), DMSP data from NGDC NOAA (https://satdat.ngdc.noaa.gov/dmsp/data/ (accessed on 16/11/2021)), and geophysical parameters data were obtained from NASA/GSFC’s Space Physics Data Facility’s OMNIWeb service (https://omniweb.gsfc.nasa.gov/ow_min.html (accessed on 16/11/2021)). The DMSP SSUSI results were obtained from the Johns Hopkins University Applied Research Laboratory (http://ssusi.jhuapl.edu/images_daily_l1b (accessed on 16/11/2021)). The AE index was provided by WDC for Geomagnetism, Kyoto (http://wdc.kugi.kyoto-u.ac.jp/ae_realtime/201808/index.html (accessed on 16/11/2021)); Kp index was provided by GFZ Potsdam (ftp://ftp.gfz-potsdam.de/pub/home/obs/kp-ap/tab/ (accessed on 16/11/2021)). GNSS data are available with UNAVCO (ftp://data-out.unavco.org (accessed on 16/11/2021)), CORS (ftp://geodesy.noaa.gov (accessed on 16/11/2021)), IGS (ftp://igs.bkg.bund.de/IGS/obs/ (accessed on 16/11/2021)), SmartNetNA (www.smartnetna.com (accessed on 16/11/2021)), Natural Resources Canada (webapp.geod.nrcan.gc.ca (accessed on 16/11/2021)), CHAIN (ftp://chain.physics.unb.ca/gps/data/daily (accessed on 16/11/2021)), SOPAC (ftp://garner.ucsd.edu (accessed on 16/11/2021)), RBMC Brazil (ftp://geoftp.ibge.gov.br (accessed on 16/11/2021)), RAMSAC CORS NGI Argentina (https://www.ign.gob.ar/NuestrasActividades/Geodesia/Ramsac/DescargaRinex (accessed on 16/11/2021)), BKGE (ftp://igs.bkg.bund.de/euref/obs (accessed on 16/11/2021)), IGN (ftp://rgpdata.ign.fr (accessed on 16/11/2021)), SWEPOS (swepos.lantmateriet.se (accessed on 16/11/2021)), SATREF (http://www.kartverket.no (accessed on 16/11/2021)), NOANET (www.gein.noa.gr (accessed on 16/11/2021)), Geosciences Australia (ftp://ftp.ga.gov.au/geodesy-outgoing/gnss/data (accessed on 16/11/2021)), and South Africa TrigNet (trignet.co.za (accessed on 16/11/2021)).
